# Development of immunohistochemistry services for cancer care in western Kenya: Implications for low- and middle-income countries

**DOI:** 10.4102/ajlm.v5i1.187

**Published:** 2016-05-04

**Authors:** Kirtika Patel, R. Matthew Strother, Francis Ndiangui, David Chumba, William Jacobson, Cecelia Dodson, Murray B. Resnic, Randall W. Strate, James W. Smith

**Affiliations:** 1 Department of Immunology, Moi University, Eldoret, Kenya; 2 Oncology Department, University of Otago, Christchurch, New Zealand; 3 Department of Pathology, Moi Teaching and Referral Hospital, Eldoret, Kenya; 4 Department of Pathology, Moi University College of Health Sciences, Eldoret, Kenya; 5 Department of Pathology, Indiana University School of Medicine, Indianapolis, Indiana, United States; 6 Histology Laboratory, Indiana University Health, Indianapolis, Indiana, United States; 7 Department of Pathology, Brown University, Providence, Rhode Island, United States

## Abstract

**Background:**

Cancer is becoming a major cause of mortality in low- and middle-income countries. Unlike infectious disease, malignancy and other chronic conditions require significant supportive infrastructure for diagnostics, staging and treatment. In addition to morphologic diagnosis, diagnostic pathways in oncology frequently require immunohistochemistry (IHC) for confirmation. We present the experience of a tertiary-care hospital serving rural western Kenya, which developed and validated an IHC laboratory in support of a growing cancer care service.

**Objectives, methods and outcomes:**

Over the past decade, in an academic North-South collaboration, cancer services were developed for the catchment area of Moi Teaching and Referral Hospital in western Kenya. A major hurdle to treatment of cancer in a resource-limited setting has been the lack of adequate diagnostic services. Building upon the foundations of a histology laboratory, strategic investment and training were used to develop IHC services. Key elements of success in this endeavour included: translation of resource-rich practices to a resource-limited setting, such as using manual, small-batch IHC instead of disposable- and maintenance-intensive automated machinery, engagement of outside expertise to develop reagent-efficient protocols and supporting all levels of staff to meet the requirements of an external quality assurance programme.

**Conclusion:**

Development of low- and middle-income country models of services, such as the IHC laboratory presented in this paper, is critical for the infrastructure in resource-limited settings to address the growing cancer burden. We provide a low-cost model that effectively develops these necessary services in a challenging laboratory environment.

## Introduction

Cancer is a leading cause of mortality in low- and middle-income countries (LMICs), already accounting for more deaths than tuberculosis, HIV and/or AIDS and malaria combined.^1,2^ According to the International Agency for Research on Cancer there were more than 600 000 new cancer cases and more than half a million cancer deaths in Africa in 2008, with a projected doubling by 2030.^3^ The need to develop an infrastructure for cancer research and care in LMICs has been recognised by various researchers.^4,5,6^ A core component of cancer infrastructure is adequate pathology services. The Breast Health Global Initiative provides excellent resource-stratified guidelines for the development of pathology services.^7^

However, development of pathology services in LMICs faces a number of challenges, including insufficient funds, lack of skilled personnel at all levels (technicians to pathologists), unavailable equipment and unreliable supply chains for consumables.^8,9,10^ Despite these challenges, there have been a number of approaches to solving the deficits in LMIC pathology services. Three primary themes characterise these efforts: the development of systems to export specimens, either pre- or post-processing, for reading and diagnosis by out-of-country pathologists; services offered in-country through volunteer pathologists from other countries, and the use of telepathology.^11^

However, there are shortfalls with each of these approaches. Specimen export faces challenges with acquisition of export permits, postage costs and risk of specimen loss during transit. In addition, long turnaround times hamper appropriate patient care.^12^ Volunteer pathology services have difficulty identifying sufficient pathologists who have time available for travel and service provision, loss of income for the pathologist and the cost of airfare, visas, and room and board. Once arrived, these experts often face a learning curve as they work with older and frequently ill-maintained equipment.^12^ Telepathology, although promising, is presently slowed by inadequate bandwidth and the cost of required equipment.^12^ Each of these approaches provides key elements to the successful development of pathology services for cancer care in resource-limited settings. These approaches depend on the development of sufficient local infrastructure and expertise.

In the absence of development of local expertise, inadequate sample preservation and preparation can render a well-resourced pathology laboratory inadequate for diagnosis. In this paper, we describe our effort to combine all three of these approaches, along with investment in local resources and extensive local training, to develop pathology services in a resource-limited setting for immunohistochemistry (IHC), which is essential for reliable cancer diagnosis, prognosis and therapeutic decision-making.^13^ This process required extensive collaboration, investments in training and the local infrastructure, as well as coordination with telepathology resources and volunteer pathologists.

## Description of laboratory development

### The Academic Model Providing Access to Healthcare programme

The Academic Model Providing Access to Healthcare (AMPATH) programme links the resources of the Moi Teaching and Referral Hospital (MTRH), Kenya’s second referral hospital, with the expertise and creativity of the Moi University School of Medicine (MUSoM) and a consortium of North American academic medical centres (Indianna University, Brown University, Duke University, Lehigh Valley Hospital Allentown, Pennsylvania, University of Notre Dame, Providence Portland Medical Center, Purdue University, University of Massachusetts, Medical School, University of Utah, School of Medicine, University of Toronto, Faculty of Medicine and University of California, San Francisco). The collective goal is to find sustainable solutions to many aspects of health and disease in western Kenya.^14,15^ AMPATH-Oncology evolved to deliver appropriate care to cancer patients presenting to MTRH and found that a major impediment to a successful cancer care programme was that pathology was limited only to morphologic diagnosis.

## Building blocks

According to World Bank data, in 2014, the population of Kenya was 44.86 million people (http://data.worldbank.org/country/Kenya#; Accessed: 21 April 2016.). At present, there are only two tertiary-care facilities in Kenya, one serving Nairobi and eastern Kenya, and MTRH, which serves the Rift Valley and western Kenya. MTRH serves a catchment area that includes half of Kenya’s population – approximately 20 million people. To start building an IHC programme, a first step was an assessment of the existing resources at MTRH, MUSoM and AMPATH. There were four general pathologists and six laboratory technicians serving in the Department of Pathology who provided diagnostic surgical pathology services to this population. Pathologists have a Masters in Medicine (MMed) qualification and are employed by MTRH and MUSoM, whereas technicians are registered members of the Kenya Technicians and Technologist Board and hold a certificate, diploma or degree. The surgical pathology laboratory at MTRH performs grossing services and processes tissue with a semi-automated tissue processor. Final diagnosis is based on morphologic assessment with haematoxylin and eosin staining.

Clinical personnel at MTRH frequently have co-appointments at MUSoM. The university also has non-clinical faculty involved in extensive laboratory and research endeavours. Relevant to IHC services, the Department of Immunology had 10 staff members, was responsible for the training of medical, dental, nursing, physical therapy and environmental health students, and had 100 m^2^ of laboratory space for training. Within the Department of Immunology, one faculty member with extensive prior experience with IHC was identified, having trained and worked in the United Kingdom, and was a certified assessor for the United Kingdom National External Quality Assurance Scheme.

AMPATH, which had been operational since 2001, had also put some infrastructure in place. Prior investment in MTRH Pathology included a tissue processor, tissue embedding station, convection oven, microtome, tissue section flotation water bath and microscopes. A critical contribution was maintaining service contracts and reagent agreements with a local company representative (DAKO, Nairobi, Kenya), which services IHC equipment and stocks reagents. This allowed for functional equipment and easy access to reagents, a frequent issue in resource-limited settings. In addition, AMPATH had extensive connections to pathologists through its large number of research projects and linked university programmes in North America. Several pathologists had travelled to Kenya to establish digital pathology training resources for MUSoM. The combined resources available for development of IHC are presented in Table 1.

**TABLE 1:** Building blocks: Existing resources within MUSoM, MTRH, and AMPATH, western Kenya, before 2009.

Resource provider	Equipment, Space	Personnel	

		Number	Position
MTRH	Service requisition infrastructure	4	Laboratory technicians
	Anatomic pathology laboratory	2	Records clerks
	Tissue processor	4	Pathologists†
	Microtome		
MUSoM–NCST	Water bath	4	Pathologists†
	Microwave	1	Immunologist with IHC experience
	Computer	2	Graduate students in immunology
	Printer	-	-
	Air conditioner	-	-
AMPATH	Semi-automated tissue processor	1	Laboratory technician providing on-site assessment and guidance
	IHC autostainer‡	4	Academic pathologists
	PT Link machine‡	-	-
	Five-headed microscope	-	-
	Refrigerator	-	-
	– 20 °C freezer	-	-
	Internet access		

AMPATH, Academic Model Providing Access to Healthcare; IHC, immunohistochemistry; MTRH, Moi Teaching and Referral Hospital; MUSoM, Moi University School of Medicine; NCST, National Council of Science and Technology.

† Four pathologists in total with shared appointments between MTRH and MUSoM;

‡ Provided by Dako North America, Inc., Carpentaria, California, United States.

## Building on the foundation

The development of the IHC service required several key components to be coordinated, purchased or developed: the physical housing of an IHC laboratory, the coordination of equipment, the organisation of the service, training of personnel and ongoing quality assessments.

**Physical housing:** Discussions between all partners (MTRH, MUSoM and AMPATH) identified space in the existing histopathology laboratory for IHC services. Renovations were required for temperature control (to ensure reagent stability and process reproducibility), air flow (to ensure safety for technicians using solvents for slide preparation) and back-up power supplies (for both the air conditioning unit and the reagent storage refrigerators).

**Coordination of equipment:** The equipment of the IHC laboratory itself was procured through repurposing of local equipment, donations and direct purchases through grants and donations. Table 2 outlines the equipment and consumables required for the IHC laboratory, along with the sources of these items.

**TABLE 2:** Strategic investments to develop immunohistochemistry laboratory services, western Kenya, 2009–2012.

Equipment	4 °C refrigerator†	IHC staining boxes‡	Tissue procesesor,§ microtome§	Microwave,† Water bath‡
Cost in USD	$1000	$400	$60 000	$550
Consumables	Disposable microtome blades‡ Tissue paper§ Coplin jars†	Coverslips† IHC slides† Slide marking pens‡ Time^r^s‡	Antibodies and staining kit†,‡ Disposable plastic ware† Forceps‡	Alcohol† Xylene† Distilled water§
Cost in USD	$1000	$2000	$10 000	$300
Personnel	Four pathology technicians were trained in IHC, along with two graduate students over a period of six months (one-week course conducted three times). Technicians were also sent to Aga Khan Hospital, Nairobi for IHC training. Kenyan pathologists were trained in reading IHC slides by pathologists from Indiana University and Brown University visiting Kenya for a period of two weeks twice a year.			

IHC, immunohistochemistry; USD, United States dollars.

† Purchased using Pfizer unrestricted grant and National Council of Science and Technology grant;

‡ Donation by Indiana University;

§ Supported by MTRH Pathology.

**Organisation of service:** Administrative structures had to be established between partners to facilitate personnel management, establish processes for requesting IHC and reporting results, and perform administrative tasks such as ordering reagents. A laboratory director was assigned from the MUSoM Department of Immunology and charged with overseeing daily operations and reporting to the head pathologist within MTRH’s histopathology department. Management of the laboratory’s finances (both expenditures and billing) was routed through an office for research and sponsored projects maintained by all three partners.

**Training of personnel:** Four histopathologists, four technicians and two doctoral students in Immunology were trained both through short group workshops conducted by the local IHC expert and representatives from DAKO, the company providing IHC reagents (Nairobi, Kenya), and through one-on-one instruction taught by the consortium of North American universities. Personnel were selected for training from amongst available staff at MTRH who were willing to take on the extra IHC training duties. Skills developed included manual staining and use of the autostainer. Pathologists were also trained on the importance of IHC in diagnosis and prognosis, and sessions with surgeons emphasised the importance of quick fixation for good IHC results. Protocols were developed to ensure that specimen transport from the operating theatre to the histopathology laboratory occurred in a timely manner.

**Quality assessment:** Following initial competency assessments by North American experts, protocols were developed to ensure ongoing quality and reproducibility. Methods used to maintain high quality included: written standard operating procedures, inclusion of positive and negative controls in all staining batches and use of electronic image transfer for consultation with North American pathologists. In addition, during routine site visits by the North American pathologists, a selection of IHC slides were assessed and stained slides were sent to reference laboratories at Indiana University and the University of California, San Francisco. Further, the IHC laboratory was enrolled in the United Kingdom National External Quality Assessment Scheme, an external quality assessment scheme, for validation and quality assurance/quality control of staining and reporting.

### The current immunohistochemistry programme: Operations and outcomes

#### Operations

Establishing a functioning IHC service started at the level of tissue acquisition. Standard operating procedures were designed to ensure that specimens are immediately placed in formalin, that the time of collection is recorded and that the attending surgeons make radial cuts across larger specimens to ensure adequate penetration of formalin. Schedules were developed for specimen transport to the laboratory and rapid grossing, followed by processing and embedding into paraffin within 24 hours of resection. A major quality improvement effort was required to ensure 3–5-µm sectioning and ultimately required gross analysis is performed by a pathologist rather than a technologist. The lead pathologist must request specific IHC stains following haematoxylin and eosin staining and reporting.

For the IHC laboratory, one pathologist is assigned for all aspects of IHC analysis, including reporting and dispatch of results. Antigen retrieval for IHC is performed with a PT link machine (Pre Treatment Link, DAKO North America, Inc., Carpentaria, California, United States). Antigen retrieval is achieved by using high-pH Tris-EDTA (pH 8.0) (DAKO K8004) and a low-pH citrate buffer (pH 6.0) (DAKO K8005). For example, high-pH antigen retrieval is used for oestrogen and progesterone receptors, whereas a low-pH antigen retrieval buffer is used for HER2.

The DAKO Envision Flex (DAKO, K8000 Denmark) kit is used for immunostaining. Endogenous peroxide is blocked by DAKO hydrogen peroxide (DAKO SM801 K8002). Ready-to-use primary antibodies are used and staining is performed as per the manufacturer’s (DAKO IR/IS series) instructions. Envision Flex Mouse linker (K 8021) is used as a secondary antibody. Slides are than labelled with Envision Flex linked with horseradish peroxidase (HRP) (DAKO SM802) and the staining is visualised by using the substrate DAB chromogen mix (DAKO SM 803). Slides are counterstained with Envision Flex Heamatoxyline (DAKO K8008), dehydrated and cover slipped, and examined under the microscope. Two Tris buffer washes of 5 min each are performed between all steps. Known positive and negative controls are also stained for all runs for quality assurance. Other immunostains used to report other tumours are shown in Table 3. The Allred scoring system is used to score the slides for the hormones.^16^ The other immunostains are reported as percentage positive or negative in the tumours. Most stains are performed manually, although used infrequently at this time, a donated autostainer (DAKO Autostainer Plus, DAKO North America, Inc., Carpentaria, California) is maintained for large-batch staining (48 slides).

**TABLE 3:** Current productivity of the MU/MTRH/AMPATH immunohistochemistry laboratory, western Kenya, 2009–2012.

Immunostain	Number of cases (cumulative to December 2012)	Frequency of testing	Scoring system	Accreditation
ER for breast cancer†	100	Once weekly	Allred score‡	UKNEQAS
PR for breast cancer§	100	Once weekly	Allred score‡	UKNEQAS
HER2-neu breast cancer¶	100	Once weekly	Negative: 0 or 1 + Borderline: 2 + Positive: 3 +	UKNEQAS
Undifferentiated tumours CD3, CD20, CD45††	52	Once monthly	Positive stain in the tumour	IU/Brown pathologist
Kaposis sarcoma LANA‡‡	35	Once monthly	Nuclear staining in tumour	UCSF pathologist
Wilm’s tumour KI-67§§	19	Once monthly	Nuclear staining in tumour	IU/Brown pathologist

**Total**	**406**	**-**	**-**	**-**

ER, oestrogen receptor; IU, Indiana University; LANA, latency-associated nuclear antigen; PR, progesterone receptor; UCSF, University of California San Fransisco; UKNEQAS, United Kingdom National External Quality Assessment Scheme.

† DAKO IR084;

‡ The score takes into account the number of cells that are positive and the intensity of staining;

§ DAKO IR068.

¶ HER2 DAKO A0485;

†† CD3, DAKO IR50; CD20, DAKO IR604; CD45, DAKO IR751;

‡‡ Bioscience CM A807;

§§ KI 67 DAKO IR626.

Once the IHC staining is complete, all staff pathologists have an opportunity to review the slides for interpretation. Three pathologists from Indiana University visited the laboratory for a two-week period twice a year and one pathologist from Brown University visited the laboratory once during the inception period. These visits provided continuous training. As per protocol, IHC slides are read together with the pathologist, IHC laboratory director and technologists. All IHC results are recorded in a logbook for technical purposes and in a file dedicated to IHC, with the matched control slide results for future reference. All slides are catalogued, dated and stored for easy reference. Paraffin blocks are also catalogued and stored, according to standard laboratory practices. Owing to constrained resources, scanning slides for electronic storage and subsequent distribution was not practical. Consequently, a photomicroscope was put in place to photograph slides in the MTRH IHC laboratory. We are adapting this approach to capture images using a mobile phone, likely the best approach in a resource-poor laboratory.

#### Outcomes

From 2006 to 2012, there was increased demand for basic histopathology – 1200 cases in 2006 rising to 6500 cases by 2012 – largely related to the clinical oncology programme. Along with this increased service demand, immunostaining provided an invaluable adjunct to diagnosis of cancer. To date, the IHC laboratory performed 406 immunostains. Before 2009, the turnaround time for results was at least three weeks, whereas after 2012 the turnaround time decreased to one week. Patients were treated as per the results obtained. The types of other immunostains performed and the method for external validation are shown in Table 3.

BOX 1 Lessons learned.

- Immunohistochemistry is required for cancer diagnosis and treatment services in resource-limited settings.
- Lack of access to equipment, maintenance requirements, and the large volume of consumables made automated approaches infeasible. Protocols and procedures that limited use of reagents and disposables allowed for a sustainable IHC laboratory to be developed.

## Discussion

Recent studies in sub-Saharan Africa have suggested improving pathology services to be key in cancer control, treatment and research.^17,18^ Building local capacity for IHC in LMICs has several tangible benefits: rapid turnaround time, improved pre-treatment diagnostic accuracy and capability for more rapid treatment initiation. Further, it avoids the risk of specimen loss associated with the use of remote laboratory facilities. Currently, the MTRH IHC laboratory is the only laboratory of its kind in a public institution in Kenya. However, similar efforts are ongoing in Malawi, which has already proven to be a robust platform for providing cancer care to its patients and for research.^19^ Currently our laboratory performs oestrogen, progesterone and HER2 staining for breast cancer treatment following guidelines,^20,21^ differentiation of lymphomas using a basic panel of antibodies,^22,23^ research-based diagnostics for Kaposi’s sarcoma (KS) using latency-associated nuclear antigen (LANA-1) and Wilm’s tumour. This work helps to facilitate treatment of patients, avoid treatment of clinically ambiguous presentations of disease as cancer^24^ and more efficiently utilise limited treatment resources.

However, in developing IHC capacity several issues were highlighted in pathology in Kenya. It quickly became clear that reliable IHC results were dependent upon good morphology, which, in turn, relied upon proper handling and fixation of tissues.^25^ Identifying simple issues, such as replacing non-standardised fixatives with neutral buffered formalin and standardising fixation, avoided issues of masked antigen, which were found to be important to allow interpretation of stains.^19^ In addition, investment in skills development of technologists was required to allow thin (3−5 μm), reproducible sections of paraffin-embedded tissues to be cut for IHC and with regard to the use and repair of the microtome. The IHC staining technique is a new technique and can be performed manually or with the use of an autostainer and the PT link machines for antigen retrieval at our institution. Several technologists had to be trained on the principles of the IHC technique, methodology and the use of equipment for IHC.

Retention of highly qualified personnel after training is an ongoing concern, owing both to personnel leaving for better employment and to internal procedures whereby technologists are rotated regularly across clinical laboratories within MTRH, a mechanism designed to ensure equity amongst technologists. This approach, common in resource-constrained settings, required specific negotiation with hospital administration and allowed ongoing skills development. Pathologists and technical staff should be trained locally, where the resources and equipment are relevant. Training should be continuous and immunostaining should be performed at least once a week for maintaining knowledge, skills and technical ability. Training of local pathologists and technologists by experts from other institutions (locally-based and internationally-based) should be encouraged. Under ideal circumstances, there should be ongoing support from experts for troubleshooting, quality assurance and external review of slide evaluation and reporting. This process required extensive collaboration, investments in training and the local infrastructure and coordination with telepathology resources and volunteer pathologists.

However, a notable lesson learnt is that not all resource-rich practices can translate to the low-resource environment. IHC performed manually was less costly and equally effective compared with the use of an autostainer. Autostaining used considerable amounts of reagents and solvents and frequent mechanical failures were experienced owing to power fluctuations. Similarly, we found that a microwave or a pressure cooker instead of expensive antigen retrieval equipment was adequate for good IHC staining in western Kenya. Given the high cost of servicing, we recommend manual batch processing of tissues for staining as the standard methodology in a resource-poor setting. Small amounts of manual staining can be performed in slide storing boxes instead of having to use expensive humidifying chambers.

Diaminobenzidine (DAB), which is a potential carcinogen, is used as a chromogen during IHC staining. Gloves and a face mask have to be worn when handling DAB. The DAB waste at our institution was diluted with water to a concentration of 0.9 mg/mL before being discarded in the hazardous waste container. The IHC laboratory is air-conditioned so there is a constant exchange of air and the temperature is maintained at 20 °C. All the stages that involved solvents such as alcohol and xylene (i.e. dewaxing, dehydrating and cover slipping) are performed in the histology laboratory, which is equipped with a fume hood.^26^

Consumables and reagents (e.g. alcohol, xylene, disposable plastic pipettes, pipette tips, cover slips and charged slides) remain a challenge to acquire and, relative to developed settings, are quite expensive. Further, there can be substantial delays in receiving purchased quality reagents. We are continuing to negotiate local purchase versus importing supplies from our international partner institutions, where competitive pricing, quality assurance and reliable delivery may outweigh the drawbacks of international importation. Despite our obvious cost constraints, we have selected more expensive ready-to-use polymer-based reagents for the following reasons: procedural simplification as a result of bound secondary antibody and a detection system which minimises errors, no reliance on avidin and biotin for conjugation and the potential for high background readings, and longer shelf stability.^20^ Our experience has been that the additional cost of buying ready-to-use reagents, which are more expensive ($200 United States dollars [USD] for 200 primary antibody tests) than the undiluted reagents ($200 USD for 300 tests), is outweighed by the benefits of obtaining consistent results, with fewer chances of repetition, in a LMIC setting.

### Conclusion

Researchers working in sub-Saharan Africa have stressed the urgent need for improvement of cancer therapy by improving pathological diagnostic capabilities as part of capacity building efforts in low-resource settings.^27^ The Global Task Force on Expanded Access to Cancer Care and Control in developing countries has recommended improving cancer diagnosis and pathology expertise.^28^ Local capacity in pathology and laboratory services therefore has to be improved. Opportunities for collaborative models involving regional and global partners should be employed as pathology services develop in LMICs.^29^

Ultimately, this multi-modal approach resulted in an efficient LMIC pathology service performing basic immunostains with the resources and expertise to facilitate expansion. Our experience illustrates that it is possible to establish extended pathology services efficiently with the help of partnership and collaboration in Kenya, a LMIC. Development of LMIC models of services such as this is critical to resource-constrained settings seeking to address the growing burden in cancer prevention, care and research.
